# Exploring Young Millennials’ Motivations for Grieving Death Through Social Media

**DOI:** 10.1007/s41347-022-00275-1

**Published:** 2022-08-26

**Authors:** Rachel King, Pelham Carter

**Affiliations:** Department of Psychology, School of Social Sciences, Birmingham City University, Birmingham, UK

**Keywords:** Social media, Grieving, Young millennial, Thanatechnology, Qualitative, Psychology

## Abstract

Past research has explored social media grief; however, the motivations for using a range of social media sites, specifically by young millennials, to grieve death fail to be explored expansively in existing thanatology research. Fourteen young millennials participated in individual semi-structured interviews, specifically questioning their motivations for using social media sites to grieve. The interviews were analysed using the thematic analysis framework identified by Braun and Clarke (2013). Four themes were generated: online influence, to announce the death, personal benefit and the hypocrisy of online mourning. The online influence theme suggests that individuals are motivated to grieve due to online influence and pressure. The personal benefit theme suggested social media present many benefits for the bereaved, including continuing bonds, which motivated them to use these platforms. The analysis also indicated that within the motivations there was hypocrisy regarding how young millennials perceive their grief posting activity when compared to others.

The emergence of social media has affected the grief process; once a private emotional response, it is now displayed by some social media users to global audiences on social media sites (SMS). Grief, like many other behaviours and emotions, is not immune to the emergence of technology, with young millennials growing up during the rise of social media, it is only natural that this age group is now dependent and accustomed to using SMS (Lenhart, 2015), resulting in all parts of one’s life now being shared online, even death (Ware, 2016). However, the reasons how this behaviour occurs in SMS require further research. Therefore, the current study will explore the motivations for why this behaviour is occurring in the young millennial population. For the purposes of this work, the younger millennial generation can be defined as being born between 1995 and 2000 (Frey, 2018).

## Death, Grief and Mourning

Experiences of death bring grief, an emotional reaction to the loss (Granek, 2010) but also mourning, the public expression of grief (Stroebe & Schut, 2008). Posting activity on SMS regarding a death experience serves the manifestation of both grief and mourning; therefore, these phrases have been used interchangeably throughout the research.

Researchers have identified many different types of grief; complicated, chronic, anticipatory, parasocial and disenfranchised grief, adding to the view that grief is complicated (Bonanno & Kaltman, 2001). The way one deals with this grief is uniquely different depending on many factors such as sex, nature of the death, religion, culture (Suhail et al., 2011) and relationship (Anderson, 2010). Interestingly, parasocial grief, the one-sided emotional reaction to a loss (for example, the death of a celebrity) is frequently seen posted on SMS (DeGroot & Leith, 2015). Similarly, disenfranchised grief (for example, the death of an ex-partner or pet) is defined as the grief not acknowledged by society (Doka, 2002). This type of grief is also expressed on SMS because online this grief can be acknowledged in a free unpoliced space (Walter, 2015). While some spaces online are arguably policed at the whims of similar social norms to face-to-face interactions if certain thresholds are met (Billingham & Parr, 2020), the nature of some online spaces allows for disinhibited sharing (Walter, 2015). Like-minded individuals and safe spaces or communities to share experiences with are often cited as motivations for online engagement across a range of areas such as gender (Clark-Parsons, 2018), sexuality (Lucero, 2017) and taboo/non-mainstream activities such as self-harm (Whitlock et al., 2006) or sex work (Carter et al., 2021).

## Theories of the Grief Process

Kübler-Ross (1970) explained there are five stages of the grieving process; denial, anger, bargaining, depression and acceptance. This theory was originally based on a narrow sample of the feelings of patients who were dying themselves. Nevertheless, the theory has been widely adopted and used by grieving individuals to navigate their grief and deal with their loss (Rando, 2000; Worden, 2018). However, this theory does not consider the role of SMS in the grieving process.

The theory of continuing bonds (Klass et al., 1996) states relationships between the bereaved and the deceased do not terminate after death. The mourner performs actions directed towards the deceased in private to continue the bond, suggesting grief is an everlasting process. This theory has been widely supported by the traditional grieving practices performed by mourners including: preserving memories of the deceased, visiting their graves, celebrating birthdays and looking through photographs (Currier et al., 2015; Klass et al., 1996). Continuing bonds via social media take many forms but often mirror traditional practices. For example, posting happy birthday to the deceased social media profile (Irwin, 2015) and setting up memorial pages (Carroll & Landry, 2010). Williams and Merten (2009) analysed 20 Facebook profiles of adolescences who had died suddenly and found adolescents used SMS to directly address the deceased to cope with the death, concluding that continuing bonds with the deceased via SMS is beneficial for the emotional recovery of the bereaved.

## Millennials and Death

The young millennial age group, born between 1995 and 2000 (Frey, 2018), incorporates the term described as emerging adulthood, which can be described as a developmental phase representing individuals from 18 to 29 years old (Arnett, 2014). This age range, of those born post 1995, is arguably one that encompasses individuals who can be considered digital natives (Prensky, 2001, 2009). A group of individuals who are well immersed in technology and have incorporated much of it into their day-to-day lives (Helsper & Eynon, 2010). Ribbens McCarthy (2007) noted that by the age of 18 years, young millennials have experienced at least one significant death. When faced with death, they feel isolated and unsure of where and how to express their grief. This is because young millennials are potentially ill-equipped with enough life experience (Hooyman & Kramer, 2006) and may not be fully socially or emotionally mature yet (Hirooka et al., 2017). The experience of online grieving in young adults was investigated by Hoffman et al. (2021), though this sample was not a young millennial sample, a group as established above that may have different experiences and responses to loss. Further exploration is needed here, and with a specifically young millennial sample.

## Social Media

SMS are interactive computer-mediated technologies which allow individuals to create personal networks that facilitate the sharing of information, thoughts and expressions via virtual communities and networks (Kietzmann et al., 2011).

As of 2018, 88% of 18–29-year-olds use social media, the highest rate of any age group (Smith & Anderson,^ 2018^); this encompasses the young millennial generation, suggesting SMS are vastly integrated into the lives of young millennials. Despite Facebook having the most number of active users, 44% of 18–29-year-olds have deleted the app, thus reducing their usage on this site (Perrin, 2018). Young millennials have increased their use of different SMS including Instagram and Snapchat (Smith & Anderson, 2018) due to their foundation as photo-sharing platforms, which encourage different motivations for use (Alhabash & Ma, 2017). Changes in use, frequency and adoption are frequent which often means that research in social media is often a snapshot of the usage at the time rather than a reflection of stable and consistent SMS use (see Perrin, 2015, compared to Pew Research Center, 2021). The increasingly present and ever changing nature of SMS, in conjunction with the highly personal nature of some SMS experiences means this is an area that requires further research in the context of experiencing grief.

## Evolution of the Grief Process

Typically in Western cultures, discussing bereavement is uncomfortable for both the bereaved and others around them, resulting in individuals grieving in private (Leonard & Toller, 2012). Highlighted by Gorer (1965) ‘one mourns in private as one undresses or relieves oneself in private, so as not to offend others’ (p. 113). Over the past decades, private grief has been criticised for its negative impact on mental and physical health, highlighted by expressive grief advocates such as Kübler-Ross (1970). Therefore, there began a shift, from private to public expressions of grief, which is beneficial for emotional recovery after bereavement (Mitchell et al., 2012). Social media has been suggested to be responsible for this shift in traditional social norms of grieving (Wagner, 2018; Walter, 2015) resulting in grief now expressed to global audiences on SMS (Brubaker et al., 2013; Ware, 2016). This new norm of public grieving exists in social media like any other social space; individually constructed and learnt through observing others (McLaughlin & Vitak, 2012).

This online grief phenomenon can be referred to as thanatechnology, the term used to describe the use of technology such as SMS to contribute to the study of Thanatology (Sofka, 1997). Walter et al. (2012) stated that the online grieving phenomenon was to be expected because social media is a social platform and death is a social event. As young millennials have grown up during the technological boom, it is only natural that they are now accustomed to using social media (Lenhart, 2015) resulting in all parts of one’s life now being shared online, even death (Ware, 2016).

## The Technological Age of Grief

Early thanatechnology work looked at the emergence of virtual memorial pages on Facebook and MySpace which appeared on the internet in 1995 (Carroll & Landry, 2010). Memorial pages can be set up on Facebook by other users, or the deceased personal profile can become memorialised by Facebook (McCallig, 2014). Memorial pages are public pages in which individuals can leave tributes, condolences and continue the bond with the deceased (Bell et al., 2015). Carroll and Landry (2010) identified that 60% of 18–25-year-olds have posted on, viewed or created a memorial page. These memorial pages provided the participants with a perceived safe and comfortable place to mourn and maintain the bond with the deceased. However, Carroll and Landry (2010) focused on Facebook and Myspace thus limiting the generalisability of the findings to young millennials, because Facebook usage has changed in intent or reduced (in frequency/intensity) in the young millennial population (Perrin, 2018), with it less likely to act the primary SMS for use.

Not only has online grieving been displayed on memorial pages, but social media users also post grief on their own personal profiles (Patton, Macbeth, Schoenebeck, Shear, & McKeown). Patton et al. (2018) examined 408 tweets posted on Twitter aimed at two gang-related deaths in Chicago to measure how young people use social media during grief. Patton et al. (2018) concluded that Twitter was used by individuals who do not have access to support systems to acquire social support to cope with the death. The conclusions made by Patton et al. (2018) have been supported and replicated by existing literature, who also identified social media to be a supportive aid for the bereaved (Carroll & Landry, 2010; Marwick & Ellison, 2012). In addition, The online social support theory (LaCoursiere, 2001) further supports the conclusion that SMS offer grieving individuals support, but it also suggests that SMS can provide coping mechanisms.

Pennington (2013) conducted 43 interviews with 18–24-year-olds focusing the questions on whether individuals would de-friend a friend on Facebook after they had died. The findings suggested that the participant’s felt deleting the deceased friend would resemble the deletion of their life. Furthermore, the interview data identified that social media is used to cope with grief and continue the bonds with the deceased. Brubaker et al. (2013) conducted 16 face-to-face interviews questioning adults grieving activity on Facebook. Brubaker et al. (2013) generated three thematic themes for why grief is posted on social media: temporal expansion, spatial expansion and social expansion. The temporal expansion theme suggests SMS’s feature of integrating content related to the past, present and future may create norms of rapidly informing others about someone’s death or reacting toward it. The spatial expansion theme suggests SMS reduction of geographical limitations may cause norms like attending a funeral via online sources. Finally, the social expansion theme suggests SMS’s unify distinct social groups through the spread of information related to the death of an individual user. However, the participant sample ranged from 24–57 years of age; this age range does not fully encompass the young millennial age group; therefore, these results cannot be generalised to young millennials. Additionally, Brubaker et al. (2013) and Pennington (2013) did not focus on the motivations of why social media is used as a platform to grieve; these findings were an additional finding of the research. The lack of directly focusing on the motivations for this phenomenon limits understanding and exploration of the online grief phenomena.

## The Present Study

This study aimed to gain a comprehensive understanding of why young millennials use SMS as a platform to grieve death. Qualitative face-to-face semi-structured interviews were therefore used to allow online grievers to express their motivations in their own words. Also, the data collection was open to all different SMS, to allow for a comprehensive understanding of the motivations around why different platforms are used, not limiting data to Facebook grief. The research question was ‘What are young millennial motivations for using social media sites as a platform to grieve death?’.

## Method

### Design

To explore the in-depth personal motivations for why individuals post grief online, a qualitative design was utilised with a phenomenological philosophical paradigm informing the research. This paradigm is essential to the current study as it recognises the subjectivity of reality and understands there are multiple perceived truths (Mayoh & Onwuegbuzie, 2015), like the many different grief experiences (Fuchs, 2018). Although the research explored individual experiences of public grief, the analysis was conducted through a thematic lens (Sundler et al., 2019). A qualitative design was utilised because it allows participants to respond in their own words, producing valid and detailed descriptions of their experiences (Glesne, 2007). Furthermore, qualitative methods explore overlooked phenomena, making them visible, because the participant-researcher relationship probes for a deeper understanding of feelings and experiences (Patton, 2002) providing rich explanatory data (Creswell, 2013).

For data collection, semi-structured interviews were chosen because while the framework is capable of pursuing motivations for detail, it is flexible; the interviewer can follow up on unpredicted topics (Kallio et al., 2016). Additionally, semi-structured interviews were chosen because of the previous research detailing the richness of data this method delivers when exploring grief on SMS (Brubaker et al., 2013; Pennington, 2013). Face-to-face interviewing also allowed the researcher to take a non-expert empathetic attitude throughout the interview (Creswell, 2013).

It is important to note that data collection and initial analysis took place prior to the current COVID-19 pandemic. While the findings still contribute to knowledge around online young millennial grief, they are analysed and discussed outside the context of COVID-19.

### Participants

A homogenous purposive sampling method was utilised to select suitable participants (Robinson, 2014). As a phenomenological lens was being applied to the analysis, a homogenous sample is required to ensure shared experiences of grief, technology use and to ensure a young millennial age range. This shared experience via a homogenous sample will allow participants to expansively answer the interview questions (Etikan et al., 2016). The inclusion criteria included: self-identifying with being active on at least one SMS and posted grief about the death of someone they knew on a SMS. Participants were required to be between 18–24 years as this age group encompasses the target young millennial population (Frey, 2018), who have the highest percentage SMS use (Smith & Anderson, 2018). In total, 14 English speaking participants were recruited from a pool of UK-based University students receiving credits for contributing to university research on the research participation scheme. No other form of compensation or monetary reward was offered. Participants age ranged from 19–24 years old; the average was 20.8 years old (SD = 1.62). This therefore meant that all the participants could also be considered emerging adults (Arnett, 2014). In terms of ethnicity and racial identity, nine participants were White British, three were Asian, one was White and Black Caribbean, and one White and Black African. A brief description of the demographic information is shown in Table 1. Names were changed to protect anonymity and ensure confidentiality.

**Table 1:** The demographic information of the participants including; gender, social media site used and relationship with the deceased

Participant name	Gender	Relationship of the deceased	Social media site used
Mary	Female	Friend	Facebook
Tina	Female	Grandad	Facebook
Rebecca	Female	Grandad	Facebook and Instagram
Jessica	Female	Brother	Facebook and Instagram
Justin	Male	Grandad	Facebook
Maria	Female	Grandad	Snapchat
Claire	Female	Grandad	Snapchat and Facebook
Charlotte	Female	Step brother	Facebook
James	Male	Friend	Facebook
Selena	Female	Grandparents and distant relatives and acquaintance	Snapchat and Facebook
Abby	Female	Family Friend and Friend	Twitter and Facebook and Instagram
Georgia	Female	Friend	Facebook
Emma	Female	Father	Facebook and Instagram
David	Male	Mother	Instagram

### Interview Guide

The guide consisted of three main topics of discussion, generated from existing literature. Brubaker et al. (2013) highlighted the importance of asking questions to inform the interviewer of the background context of the death, including the age of the participant and the relationship they had with the deceased, as this may affect the motivations for posting; these were adapted for the current study. Pennington (2013) questioned how helpful social media was during the loss, and elements of their suggested schedule were to encourage participants to reveal how they used social media to grieve.

### Procedure

Participants signed up to participate in the study on a website platform open to social science students who attended a university. The website presented the information sheet detailing the nature of the study, and the inclusion criteria were presented. Written consent was obtained before the audio-recording started, as all participants were required to sign the consent form. Face-to-face semi-structured interviews were chosen because they allow the researcher to terminate the interview if the participant seems distressed from recognising social cues (Elmir et al., 2011; Kallio et al., 2016). Participants were asked if they are happy to continue periodically during the interview; no interviews were terminated. All interviews were audio-taped on a HOMDER TF-30 digital voice recorder, which was stored on a password-locked university Microsoft OneDrive only available research team. After the interviews, all participants were fully debriefed, informed again of their ethical rights and given helplines details.

### Analysis

The criteria outlined by Lincoln and Guba (1985) was followed to ensure the analysis was rigorous. Braun and Clarke’s (2013) guide for thematic analysis was followed to ensure good practice. Firstly, all interviews were transcribed true verbatim (Poland, 1995), then the audio-tapes were destroyed. The transcripts were analysed using an inductive thematic analysis framework which can be underpinned by phenomenology, allowing the researcher to identify rich and detailed data-driven themes (Braun & Clarke, 2006) essential for understanding under-researcher phenomena. Furthermore, this method has been used by previous thanatology research (Brubaker et al., 2013), as it is useful for examining different perspectives and unanticipated insights (Cassell & Symon, 2004).

Following the familiarisation and transcription process, codes were developed. Open coding was used, meaning the initial codes were developed and changed throughout the coding process. As a result, 91 initial codes were generated, the codes were reviewed to categorise the central concepts, then actively generated into overarching proto-themes. The themes were reviewed and refined in line with the thematic analysis procedure (Braun & Clarke, 2006). After further review, four final themes were deemed to reflect the data accurately.

## Results

Participants reflected on the motivations for why they posted grief on a range of social media platforms after the death of someone they knew. Through the thematic analysis framework, four themes were generated: online influence, to announce the death, personal benefit and the dissonance of online mourning.

### Online Influence and Social Norms

Online peer and social network influence was a strong theme running throughout the majority of the interviews. James stated, ‘all my friends we were all influencing each other […] everybody was doing it everybody when somebody died would just post on Facebook’. James suggests posting grief on SMS when someone has died is the social norm, because individuals in his social circle were all doing it. Furthermore, he stated all his friends were influencing each other implying he was also influenced by other users’ actions. This idea that individuals are motivated to post grief because of witnessing others who publicly grieve, is further supported by Claire, ‘I think it's definitely been like following on from what other people do […] I’ve seen other people posting about grief’, and also Tina, who says, ‘everyone is on Facebook now and like so like you’re tryna be like there and like do what they do so I guess yeah maybe because others do it maybe I did it’. Thus, suggesting online grieving norms exists due to observing others.

This new norm is highlighted further by the many participants who viewed this phenomenon as normal. Charlotte stated, ‘like I said it was quite natural so people didn’t really question it really it was just what happened when something major happened in your life you share it’. Maria similarly perceived that her grieving on Snapchat was normal, by stating, ‘I dunno I just thought that’s what you’re supposed to put’, supporting the view that death is an event to be experienced with an expectation of expressing a social platform. In addition, Mary whose friend died of suicide said, ‘I think that sister was the only one at the time she didn’t say anything about it’*,* implying it was weird that the sister did not post and her actions did not follow the online grieving norm.

Interestingly participants also picked up on an element of pressure to post their grief and Selena even felt ‘compelled to post’. James detailed that ‘everyone was doing it you kind of feel like you have to do it as well coz you want to be a good friend people would think you don’t really care that much if you don’t post’. James implies an element of pressure motivating him to post, in fear that neglecting to express his grief online reflects that he does not care for the deceased. Maria likewise stated, ‘I feel like if you didn’t post it people would just think oh it's fine she wasn’t that close to her anyway’*.* This suggests part of the motivation for posting is derived from avoidance of appearing not to care. The current results also suggest individuals are motivated to post grief on different SMS, not just Facebook, due to the new norm of online grieving, which has created a pressure to publicly express that they cared for the deceased. In this case, Maria was referring solely to their experiences using Snapchat as they were a non-Facebook user, and Selena also used Snapchat. Social media appears to facilitate this expression of love and care towards the deceased, but social influence is motivating young millennials to post online regardless of whether this is Facebook or alternative platforms.

### To Announce the Death

Announcing the death was also a common reason for posting grief online. Social media is defined as an information sharing site; therefore, it is perceived as a tool for this purpose, supported by Emma’s ‘Facebook is more about sharing information’. Several participants who were family or close family friends of the deceased reported that SMS was used to announce the death to their followers. Emma and her family set up a page on Facebook for her father who was missing, when later found dead, the family changed the missing page to a memorial page ‘and then um posted a picture and like a paragraph just to let everyone know it it was the easier way to let a massive amount of people know’. Here, Emma suggests that the reason for posting grief online was to announce his death to the masses of people who were helping search for him, and social media was the easier way to do this. David similarly referred to using SMS for this reason:

I was out for a meal and a really old friend we bumped into him and he said how’s your mum and it's a bit awkward when you have to tell them she’s passed away I feel like this post will just let people know who doesn’t know.

David is intending to post another post on social media to inform others his mother has passed. Furthermore, one participant in particular, Rebecca, stated that not only did she use SMS to announce the death but also to post information about the memorial service ‘so first thing I posted when he passed away was obviously the funeral details’. One explanation for this finding may be due to social media’s temporal expansion, which suggests SMS integrate past, present and future which create a norm of informing others of the death. SMS platforms have both synchronous and asynchronous elements stretching the temporality of information sharing. A post here may be accessed soon after death, or weeks and month later. Grieving can be revisited at various points and practical information accessed without direct and real time interaction.

Interestingly, participants referred to using different SMS to announce the death due to the different audiences. Claire explained, ‘like for Facebook family members would see it…’. Whereas Selena suggests, ‘Instagram wasn’t as big and it was only my friends’. Similarly Maria posted on snapchat, ‘cuz it had my friends’. This shows why different platforms might have been used when grieving, to let different audiences know of the death. Another potential consideration here is that millennials may have different audiences on different platforms due to issues around privacy or the self-identity they are wishing to express. More candid grief may be displayed to more intimate audiences for example on SMS that host those individuals. A different persona, identity or reaction might be presented on platforms where individuals worry about greater observation or surveillance, for example platforms that also include family members as well as friends may be utilised differently. This is an area for further exploration.

### Personal Benefit

The theme of posting grief online for personal benefit was a motivation for using SMS in all participants, but these personal benefits were represented in many different forms. Many participants referred to SMS as a platform to gain support, including Abby, ‘I think I just wanted a bit of support’ also expressing this further:

I think because other people had done it and they got support as well that’s why I felt like I could too […] I’ve seen other people get support like I’ve reached out to people that posted so I kind of thought people would do that for me.

This quote not only shows the interlinking aspects of the influences from observing others but also highlights SMS are viewed as a social support platform, wherein support can be gained during grief. Likewise, Tina suggested gaining support via SMS motivated her to post, ‘people can relate to you […] if they have been through something similar they can encourage you sort of thing like it's just seeking for help kind of thing’. Thus, SMS allow grieving individuals to make connections with people who have experienced similar grief, providing them with help and support to cope; they are viewed as an inclusive community.

Another reason for posting grief online is that SMS allow participants to manage their grief behind a screen, which is beneficial, as some participants disclosed they were not comfortable with expressing their grief in person. Georgia explained:

I just find it hard to say my feeling to people and so social media was easier because I didn’t need to actually tell people face to face how I felt […] it was easier to post because you are behind a screen type thing.

Similarly, Selena explained, ‘ like I can say what I'm feeling on a screen and then everyone else can just like interpret it rather than them coming up to me and ask me questions I'm not comfortable to answer face to face yet’. This normalising of indirect grief sharing may be particularly relevant for young millennials who find it difficult to share feeling in a face-to-face environment.

### Sub-theme: Expressing Feelings and Emotions

SMS were used in order to share feelings and emotions towards the death; such expressions are beneficial for emotional recovery after bereavement. Tina referred to Facebook as somewhere ‘you are free to express how you feel’; this view was also shared by many other participants. Tina referenced Facebook as a ‘form of diary’, which ‘helps you release emotions rather than just keep it in’, implying Facebook is viewed as a platform to write down and release ones emotions, much like the purpose of a diary.

Justin further explained posting grief on SMS is:

a sense of release very temporary […] I felt like the tiniest bit better just to know that I'm venting it out just to not keep it all in me even […] I’ve like said it I’ve written it down basically and just put it out there and just kind of thrown that out of me obviously I still feel shit I still feel upset but it's not like building up.

This release of emotions facilitated by SMS, highlighted by Justin, results in a cathartic release, which is potentially beneficial for grieving individuals. Therefore, suggesting SMS also offers a cathartic release for individuals grieving the loss of someone they knew.

Furthermore, one participant in particular, Charlotte, explained expressing her emotions on Facebook allowed her to accept the death and have closure ‘I think it was my way of accepting it if it's out there if it public if everyone know I just have to accept it’. This quote illustrates the denial and acceptance stages of the five stages of grieving. Therefore, SMS may facilitate traditional stages of grief rather than act as a new stage or element.

Many participants even explained that SMS are their only outlet for expressing their emotions. James said, ‘I was really sad so I didn’t know what else to do like what do I do I don’t talk to my parents about this I don’t talk to anybody’ and later explained ‘I just don’t like talking to people about this stuff’, showing that James does not talk to anybody about his feelings and does not like speaking in person about the death. Similarly Maria stated, ‘I don’t think I'm the type of person to sit with someone and talk about my feelings it's weird to me so I just post it’. These quotes are suggestive of feelings of isolation or disconnection from others, with SMS acting as what is seen as an acceptable and appropriate outlet for the expression of grief.

An interesting point arose from two participants Emma and Mary who were grieving a death through suicide. Both participants suggested the reason they posted grief online was to reduce the negative stigma attached to suicide. Emma said:

with suicide you almost want to make a point like he was this this and this because you don’t want that stigma attached with it and you want to talk about it and be open about it and you’re not afraid to say that this is how he died so I think that’s why I posted as well because I want to be as open about it and social media is a good way of reducing stigmas to get word out and stuff.

Emma expressed her feelings of her father online to reduce the negative stigma of suicide in general but also to detach the stigma that may be placed on him. Similarly, Mary stated,‘I just felt like I had to share her and how nice she was and that she was a nice person’, showing Mary felt the need to post positive properties about the girl. Further research should be conducted on this specific type of death with specific prompts to determine if this theme is prominent for the bereaved through suicide. This is potentially an example of disenfranchised grief. With the social norms around discussing suicide, the ability to grieve publicly and how this is received may differ. As the participants noted, there is a stigma, and such grief may often end up disenfranchised if not expressed online in this manner to challenge the stigma.

### Sub-theme: Continue Bonds with the Deceased

This theme was presented throughout the majority of the interviews, as many participants explained they posted pictures, memories, videos and statements to continue bonds with the deceased. Jessica explained her posts were directed towards her younger brother who died, ‘I miss you so much’. Emma explained this behaviour of directing the post at the deceased, ‘you feel like you want to keep him alive so you carry on posting’. Similarly, Georgia explained, ‘when you post I guess it feels like you are talking to them […] it just feels like you are so it makes you feel closer to them’. These findings suggest attempts to directly address the deceased, maybe in an attempt to continue their bond. The current findings also suggest this practice is not limited to Facebook, and it is also presented on other SMS such as Instagram. Therefore, continuing bonds with the deceased is not limited not only to private practices but also public displays.

Additionally, the analysis highlighted that SMS allow grievers to mirror traditional grieving practices of continuing bonds. Emma said, ‘Facebook now has like the biggest online cemetery so in a sense you’re not speaking to the grave but you sort of are’, suggesting SMS mirror traditional practices of visiting a grave site to continue the bonds. The participants also explained they posted directly at the deceased on birthdays and death anniversaries; Georgia explained this was for the purpose of ‘wishing her a happy birthday’, which mirrors traditional practices of continuing bonds but displays them overtly on SMS.

A notable difference in the current results when compared to existing research is that some participants viewed their sharing of memories, photos and statements regarding the deceased were for the benefit of others who viewed the post and not for their benefit to continue bonds. Jessica explained posting memories to remind people of her brother:

so the reason we still do kind of post is to get people remember[…] to remember like who he was because obviously he was only young […] a lot of his friends you know don’t really remember him but it's just to kind of get people to remember who who he was obviously posting pictures your reminded of what he looked like.

### The Dissonance of Online Mourning

There was a dissonance within the motivations of some participant’s interviews when reflecting on their motivations versus the perceived motivations of others. Some participants viewed their online grief as legitimate but viewed others negatively as attention seeking. Furthermore, the participants justified their actions with their young age at the time of posting to deal with the cognitive dissonance.

Charlotte stated, ‘I find it bit of a attention seeking in a way’ and later explained ‘social media had created this idea of you only post the best things […] so when somebody would put out something so negative and such a hard experience it's seems like you know they want sympathy’. Furthermore, David viewed other people’s online grief as ‘it's just sort of their making their death about you like attention seeking from their death’ but views his own motivations as to announce the death of his mother. This highlights the hypocritical views of people who even post grief themselves.

James in particular makes reference to the cognitive dissonance, ‘it was a really kind of fighting with my own beliefs at that time as well like even back then I didn’t like attention seekers’, showing James was conflicted; he posted his grief online even though he was fighting his own beliefs. But later, James stated viewing other people’s motivations as different to his:

I see a lot now I know a lot of people now who have posted for attention and it really pisses me off coz you can’t use somebody’s pain for your own good in life for your own ego I know people who do it a lot and it's not okay.

This revelation suggests James was experiencing some form of cognitive dissonance or conflict between personal expectations and that of others. James with hindsight justified his behaviour, by changing his cognitions, ‘I just feel like when you’re young you don’t really know how to deal with those things’. This quote highlights that James may have justified his grief posts by believing his young age determined his online behaviour to reduce the dissonance felt.

This theme highlights that knowledge and connection do not produce sympathy or empathy. This was an unexpected finding. In the future, the dissonance of social media mourning should be explored in depth.

## Conclusion

The current study identified the motivations for why SMS are used as a platform to grieve death in the relatively under researched sample of young millennials. The themes have built on the limited understanding of the motivations for this phenomenon.

When considering the theme of social norms, our findings suggest online grieving norms exists like any other norm, due to the online observation and surveillance of others and the expectation that such observation is reciprocated (McLaughlin & Vitak, 2012). Our findings supported the view that death is a social event and expressing this on a social platform is to be expected (Walter et al., 2012). The findings around the use of SMS to demonstrate that young millennials cared for the deceased supported and extended the work of Egnoto et al. (2014), to include platforms other than just Facebook.

The theme of announcing death supported to an extent the notion that SMS offer a distortion to the temporality around death (Brubaker et al., 2013). As a medium with the potential for asynchronous interaction, both practical and grief-based interactions can be stretched over a long-time period. Indeed, the nature of SMS affords persistence to death and memorials of death. The dead live on digitally (Chen, 2012), and the interaction with those individuals has a persistence that can be flexible and negotiable on an individual basis (Marwick & Ellison, 2012). As viability differs, then engagement with such memorials can differ, especially dependent on audience.

It was suggested within the personal benefit theme that SMS allows grieving individuals to make connections with people who have experienced similar grief, providing them with help and support to cope; they are viewed as an inclusive community (Dawson, 2006). The overwhelming view that SMS offer support enhances the creditability of the online social support theory (LaCoursiete, 2001) which proposes SMS can be used to gain social support and coping mechanisms. As highlighted before, young millennials often feel isolated and unsure of how and where to grieve when faced with death (Ribbens McCarthy, 2007). This isolation causes a need for support, as they are not equipped with enough life experiences (Hooyman & Kramer, 2006), and this theme suggests that SMS fill the void and provide support. Egnoto et al. (2014) suggested, since SMS have become socially accepted, individuals have become more comfortable with online grieving, and the current findings suggest this is particularly relevant for young millennials who find it difficult to share feeling in a face-to-face environment.

The expressing emotions and feelings sub-theme highlighted that social media facilitate the five stages of the grief process (Kübler-Ross, 1970) rather than act as a sixth stage. Thus, social media should be incorporated to allow this theory to be applied to modern grieving practices. The in-depth findings here mirror the analysis of open-ended questionnaire responses from Hoffman et al. (2021). Also, the findings suggest the theory of continuing bonds (Klass et al., 1996) is temporally biased and requires SMS to be recognised as a way to facilitate continuing bonds for younger grievers.

In particular a sub-theme of continuing bonds was developed. This theme was presented throughout the majority of the interviews, as many participants explained they posted pictures, memories, videos and statements to continue bonds with the deceased, which is a beneficial practice for the mourners’ emotional recovery process (Williams & Merten, 2009). Additionally, these findings are consistent with existing research, concluding Facebook is used to continue bonds with the deceased (Carroll & Landry, 2010; Egnoto et al., 2014; Pennington, 2013) and relates to early discussion of temporality and visibility online. This is suggestive of traditional practices being overtly present on SMS (Irwin, 2012). Klass et al. (1996) referred to continuing bonds as behaviours and actions directed at the deceased rather than at others. However, whether participants were posting grief for the benefit of their followers or whether it was to continue bonds with the deceased by proxy remains to be seen.

The current study provides some further unique contributions to thanatological research and young millennial grief. Specifically the inclusion of feelings of dissonance of online mourning with the theme of dissonance extends recent findings from Moyer and Enck (2020). Moyer and Enck (2020) did find similar themes around motivations for posting grief on SMS (such as making connections and general expression) but despite considering the posting of others did not find similar dissonance. They found that the posting of grief by others was seen as normalising grief or acting in a cathartic manner. Some conflict was noted, but this referred to the discomfort of witnessing grief rather than the grief being considered performative and negative. Within this work there is a disconnect between motivations attributed to the individual and those attributed to others with a positive to negative split, but this distinction is difficult as we have to consider whether declarations of grief are performative or not (Dobler, 2009; Marwick & Ellison, 2012).

The findings also hold important practical implications. The personal benefit theme shows that young millennials are motivated to use SMS to gain support because they feel comfortable using SMS to grieve. Therefore, the healthcare sector must consider the possibility that SMS are the new way to provide support, because while there are grief resources available on the internet, young millennials also turn to SMS for grief support. This phenomenon will continue to grow as the prevalence of SMS and the online grieving norm increase; therefore, social workers, grief counsellors and psychologists should consider the development of support programs utilising SMS to support these individuals. This is vital because the negative effects associated with bereavement for example, clinical depression (Shear et al., 2011) and suicide (Szanto et al., 2006) are linked with a lack of support (Shear, 2015); therefore, it is vital the healthcare sector incorporates the use of SMS to remain current to reduce these negative effects of bereavement in young millennials.
